# The lantibiotic mersacidin is a strong inducer of the cell wall stress response of *Staphylococcus aureus*

**DOI:** 10.1186/1471-2180-8-186

**Published:** 2008-10-23

**Authors:** Peter Sass, Andrea Jansen, Christiane Szekat, Vera Sass, Hans-Georg Sahl, Gabriele Bierbaum

**Affiliations:** 1 Institute of Medical Microbiology, Immunology and Parasitology (IMMIP), University of Bonn, Sigmund-Freud-Str. 25, 53105 Bonn, Germany

## Abstract

**Background:**

The lantibiotic mersacidin is an antimicrobial peptide of 20 amino acids that is ribosomally produced by *Bacillus *sp. strain HIL Y-85,54728. Mersacidin acts by complexing the sugar phosphate head group of the peptidoglycan precursor lipid II, thereby inhibiting the transglycosylation reaction of peptidoglycan biosynthesis.

**Results:**

Here, we studied the growth of *Staphylococcus aureus *in the presence of subinhibitory concentrations of mersacidin. Transcriptional data revealed an extensive induction of the cell wall stress response, which is partly controlled by the two-component regulatory system VraSR. In contrast to other cell wall-active antibiotics such as vancomycin, very low concentrations of mersacidin (0.15 × MIC) were sufficient for induction. Interestingly, the cell wall stress response was equally induced in vancomycin intermediately resistant *S. aureus *(VISA) and in a highly susceptible strain. Since the transcription of the VraDE ABC transporter genes was induced up to 1700-fold in our experiments, we analyzed the role of VraDE in the response to mersacidin. However, the deletion of the *vraE *gene did not result in an increased susceptibility to mersacidin compared to the wild type strain. Moreover, the efficacy of mersacidin was not affected by an increased cell wall thickness, which is part of the VISA-type resistance mechanism and functions by trapping the vancomycin molecules in the cell wall before they reach lipid II. Therefore, the relatively higher concentration of mersacidin at the membrane might explain why mersacidin is such a strong inducer of VraSR compared to vancomycin.

**Conclusion:**

In conclusion, mersacidin appears to be a strong inducer of the cell wall stress response of *S. aureus *at very low concentrations, which reflects its general mode of action as a cell wall-active peptide as well as its use of a unique target site on lipid II. Additionally, mersacidin does not seem to be a substrate for the resistance transporter VraDE.

## Background

Lantibiotics form a particular group among the antimicrobial peptides (AMPs) and are characterized by unique structural features. These result from extensive posttranslational modifications that yield the ring forming thioether amino acids lanthionine and/or 3-methyllanthionine. The ring structures contribute to enhanced resistance towards proteolysis [1] and to increased tolerance to oxidizing conditions [2]. In fact, the designation "lantibiotics" is derived from "*lan*thionine containing an*tibiotics*". Lantibiotics are produced by and act against Gram-positive bacteria and exert multiple modes of action like pore formation and/or inhibition of cell wall biosynthesis [3-5]. Mersacidin is the smallest lantibiotic known so far (1825 Da) and is produced by *Bacillus *sp. strain HIL Y-85,54728. It is an uncharged molecule of 20 amino acids forming four intramolecular thioether bridges, which confer a globular structure to the molecule [6]. Mersacidin inhibits the transglycosylation reaction of cell wall biosynthesis by complexing the sugar phosphate head group of the peptidoglycan precursor lipid II, thereby using a target binding site that is different from any other clinically used antibiotic [7]. It has been shown to successfully inhibit the growth of Gram-positive bacteria including methicillin-resistant *Staphylococcus aureus *strains (MRSA) *in vitro *and *in vivo *[8-10].

The glycopeptide vancomycin has been the antibiotic of choice to treat infections with MRSA and acts by binding the D-alanyl-D-alanine terminus of the peptide side chain of lipid II. Considering the emergence of vancomycin intermediately resistant *S. aureus *strains (VISA) since the late 1990s [11], new effective treatment strategies for MRSA are urgently needed. In this context, lantibiotics could represent alternatives for clinical applications [4] and mersacidin might be a blueprint for the development of new antibiotics to control nosocomial infections [7,9].

Previous gene expression studies of Kuroda *et al*. and Utaida *et al*. concerning the *S. aureus *responses to the cell wall-active substances vancomycin [12], oxacillin, bacitracin and D-cycloserine [13] identified a cell wall stress stimulon, which seems to be predominantly regulated by the VraSR two-component regulatory system (TCRS). Members of this stimulon comprise the *vraSR *genes together with genes related to the cell wall metabolism of *S. aureus *like *murZ*, *uppS*, *bacA*, *pbp2*, *sgtB *and genes related to protein metabolism. Hence, the VraSR TCRS has been shown to positively regulate the important steps of the cell wall biosynthesis pathway as a response to cell wall stress [12]. Recently, McAleese *et al*. described a core cell wall stress stimulon of 17 genes by merging their own results, that had been recorded in the presence of vancomycin, together with the results of Kuroda *et al*. and Utaida *et al*. [12-14]. In conclusion, the cell wall stress stimulon is characterized through a comprehensive response that involves manifold cellular processes. This general cell wall stress response seems to be conserved among Gram-positive bacteria [15]. Most recently, the transcriptional profile of the *S. aureus *response to the lantibiotic nisin has become available [16]. Nisin acts by lipid II-based pore formation and inhibition of cell wall biosynthesis [17]. The *S. aureus *response to nisin (~3 × MIC) was relatively moderate and an induction of the cell wall stress stimulon was not observed.

In our study, we used gene expression profiling by employing full genome *S. aureus *microarrays and quantitative Real-Time PCR (qRT-PCR) techniques to elucidate the transcriptional response of *S. aureus *to subinhibitory concentrations of mersacidin. To this end, we employed three *S. aureus *strains providing varying susceptibility characteristics, namely the vancomycin- and methicillin-susceptible (VSSA/MSSA) strain *S. aureus *SG511 as well as the heterogeneous VISA/MRSA strain *S. aureus *SA137/93A and the closely related VISA/MSSA strain *S. aureus *SA137/93G [18,19]. Since self-protection against mersacidin is mediated by an ABC-transporter in the producer strain, we also assessed the role of the ABC transporter VraDE, which was upregulated during exposure to mersacidin, to counteract the action of mersacidin by employing a *vraE*-defective mutant of strain SG511 [20]. We further examined the influence of cell wall thickness on the activity of mersacidin, because a thickened cell wall is proposed to be one of the key resistance mechanisms to vancomycin [18,21,22].

## Results and Discussion

### Susceptibility of *S. aureus *SA137/93A, *S. aureus *SA137/93G and *S. aureus *SG511 to mersacidin

**Figure 1:**
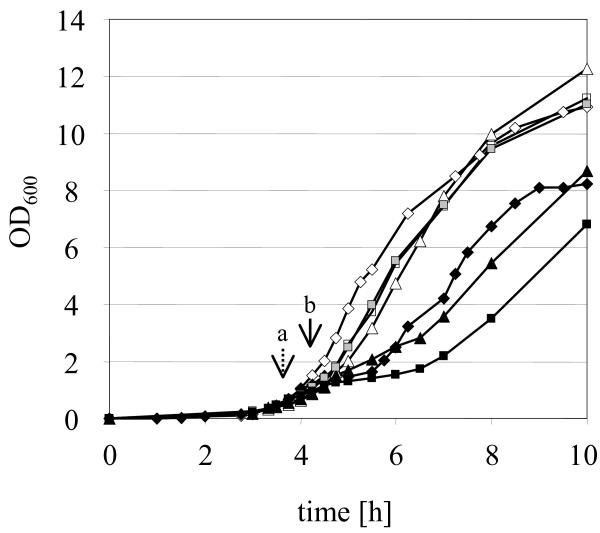
***In vitro *growth of *S. aureus *in the presence of mersacidin**. To assess suitable mersacidin concentrations for microarray experiments, the lantibiotic was added to an exponentially growing *S. aureus *culture at an OD_600 _of ~0.5 (time point "a"). In the microarray experiments, the cells were harvested at an OD_600 _of ~1 (time point "b") before the reduced growth rates of the cultures became visible. *S. aureus *SG511 (black diamonds, 1 μg/ml; white diamonds, control); *S. aureus *SA137/93A (black triangle, 16 μg/ml; white triangle, control); *S. aureus *SA137/93G (black square, 16 μg/ml; grey square, 4 μg/ml; white square, control).

The three strains displayed considerable differences in their susceptibilities to mersacidin. While the growth of *S. aureus *SG511 was already inhibited by 1 μg/ml mersacidin in BHI medium, the minimal inhibitory concentrations (MIC) of mersacidin against SA137/93A and SA137/93G were 35 μg/ml and 30 μg/ml in BHI broth, respectively. Notably, the growth retardation occurred 1 hour after addition of the lantibiotic (Fig. 1).

### Transcriptional response of *S. aureus *in the presence of mersacidin

*S. aureus *SA137/93A and SA137/93G were grown in BHI broth to an OD_600 _of 0.5, then subinhibitory concentrations of mersacidin (16 μg/ml, 0.5 × MIC) were added. Samples were taken 30 minutes (OD_600 _of ~1) after addition of mersacidin, before the decreased growth rate became apparent (Fig. 1). *S. aureus *SG511 was grown with 1 × MIC of mersacidin to induce a mersacidin-dependent transcriptional response, since the profile of the growth curve of *S. aureus *SG511 at 1 × MIC was comparable to that recorded for the VISA strains at 0.5 × MIC (Fig. 1).

Gene expression profiles of the mersacidin-treated *S. aureus *strains were compared to non-treated control cells and displayed extensive changes in gene expression. These changes were similar for all three tested strains and involved the transcript levels of 380 genes that were significantly changed (> 2.5-fold) upon mersacidin treatment in at least one of the tested strains, with 207 genes exhibiting increased transcript levels (see Additional file 1) and 173 genes showing decreased transcript levels (see Additional file 2).

### Mersacidin strongly induced the cell wall stress response of *S. aureus*

In the presence of mersacidin, the induction of the VraSR-triggered cell wall stress stimulon was one of the most striking events of gene regulation (see Additional file 1 and Additional file 2) and included 71 genes of the 161 cell wall stress stimulon members [13]. Similarly, 35 genes out of the 46 genes of the VraSR regulon [12] were upregulated after exposure to mersacidin (Table 1). Furthermore, 16 out of the 17 members of the core cell wall stress stimulon [14] were differentially expressed after mersacidin treatment.

**Table 1:** Significantly regulated genes of the VraSR-dependent cell wall stress response [12] in this study^1^

N315 ORF	Gene	Gene product	Product function	Fold change^2^			
				
				SA137/93A	SA137/93G		SG511
				(0.5 × MIC)	(0.5 × MIC)	(0.15 × MIC)	(1 × MIC)
SA0531	*proP*	proline/betaine transporter homologue	Protein transport and binding	**2.4**	**2.7**		0.6
SA0536		hypothetical protein		**30.2**	**17.1**	**8.5**	**26.6**
SA0608		hypothetical protein		**2.7**	**3.8**		**9.2**
SA0825	*spsA*	type-I signal peptidase	Protein secretion	**2.2**	1.4		**3.9**
SA0909	*fmtA*	FmtA, autolysis and methicillin resistance-related protein	Pathogenic factors	**9.3**	**3.8**	**6.3**	**6.3**
SA0914		hypothetical protein, similar to chitinase B		**14.2**	**3.9**		**15.3**
SA0931		hypothetical protein		**4.9**	**3.2**		**6.2**
SA1183	*opuD*	glycine betaine transporter	Protein transport and binding	0.9	**0.5**		**0.5**
SA1255		PTS system, glucose-specific enzyme IIA component	Protein transport and binding	**4.7**	**10.7**	**2.6**	**13.1**
SA1282	*recU*	recombination protein U homologue	DNA recombination	**3.6**	2.5	2.2	**2.5**
SA1283	*pbp2*	penicillin-binding protein 2	Cell wall related genes	3.4	2.3	2.7	**3.5**
SA1476		hypothetical protein		**11.5**	**11.3**	**6.5**	**15.9**
SA1548		hypothetical protein, similar to acylglycerol- 3-phosphate-O-acyltransferase homologue	Metabolism of lipids	0.7	**0.5**		0.6
SA1549		heat-shock protein homologue, similar to serine proteinase	Adaptation to atypical conditions	**7.1**	**4.6**	**3.9**	**5.6**
SA1657		conserved hypothetical protein		1.3	**2.3**		0.9
SA1659	*prsA*	peptidyl-prolyl *cis/trans *isomerase homologue	Protein folding	**10.8**	**18.1**	**10.0**	**33.3**
SA1691	*sgtB*	hypothetical protein, similar to penicillin-binding protein 1A/1B	Cell wall related genes	**13.1**	**8.8**	**4.8**	**9.7**
SA1700	*vraR*	two-component response regulator	RNA synthesis – Regulation	**13.3**	**8.0**	**7.3**	**17.3**
SA1701	*vraS*	two-component sensor histidine kinase	Sensors (signal transduction)	**11.7**	**8.9**	**5.8**	**17.0**
SA1702		conserved hypothetical protein		**10.4**	**9.4**	**4.4**	**12.2**
SA1703		hypothetical protein		**31.6**	**24.8**	**6.8**	**45.4**
SA1711		hypothetical protein, similar to DNA-damage inducible protein P	DNA replication, modification, repair	2.2	**1.6**		**3.4**
SA1712		conserved hypothetical protein		**24.7**	**21.0**	**5.0**	**29.4**
SA1926	*murZ*	UDP-N-acetylglucosamine 1- carboxylvinyl transferase 2	Cell wall related genes	3.2	**4.4**	3.9	**6.1**
SA2103		hypothetical protein, similar to *lyt *divergon expression attenuator LytR	RNA synthesis – Regulation	**6.4**	**6.1**	4.4	**8.0**
SA2113		hypothetical protein		**14.9**	**11.8**	**4.9**	**11.4**
SA2146	*tcaA*	TcaA protein		**3.3**	1.6	**2.2**	**4.0**
SA2220		conserved hypothetical protein		**7.0**	**12.2**	**3.7**	**21.4**
SA2221		hypothetical protein		**25.6**	**18.3**	**5.2**	**42.7**
SA2222		hypothetical protein, similar to TcaB	Protein transport and binding	1.2	**0.6**		1.0
SA2296		hypothetical protein, similar to transcriptional regulator MerR	RNA synthesis – Regulation	**8.1**	**10.4**	**3.9**	**5.5**
SA2297		hypothetical protein, similar to GTP-pyrophosphokinase	Nucleotide, nucleic acid metabolism	**9.1**	**14.1**	**3.9**	**10.3**
SA2298		conserved hypothetical protein		1.8	**2.2**		1.9
SA2413		sulfite reductase flavoprotein (NADPH)	Metabolism of sulfur	1.7	2.2		**2.3**
SA2480	*drp35*	drug responsive protein 35		2.7	**3.4**	**2.8**	**2.8**

^1 ^Significant changes of gene expression were determined by implementing SAM (significance analysis of microarrays; ) [37].

^2 ^Fold change in transcript level indicated as mean of the "median of ratios" compared to control cells. Fold change in bold = classified as "significantly" regulated in this strain by SAM.

The *vraSR *genes and several cell wall biosynthesis genes were strongly upregulated, which included the putative monofunctional glycosyltransferase gene *sgtB*, the transpeptidase/transglycosylase gene *pbp2 *and the UDP-N-acetylglucosamine 1-carboxylvinyl transferase gene *murZ*, as well as *tcaA *and *drp35*. The predominant induction of the glutamate (*gltB*, *rocA*) and lysine (*asd*, *dapAB*, *dhoM*, *lysA*) amino acid biosynthesis pathways might also support cell wall biosynthesis, since glutamate and lysine represent essential components of the peptidoglycan precursor lipid II. In fact, the disruption of the *dap *operon or the *lysA *gene has been shown to be involved in a decrease of oxacillin resistance and growth attenuation of *S. aureus *[23,24]. Especially in strain SA137/93A, mersacidin treatment induced the transcription of the *bacA *and *uppS *genes, which are involved in lipid II carrier regeneration and synthesis. Expecting the carrier level to be a limiting factor when lipid II is blocked by mersacidin, an enhanced availability of carriers might be beneficial for peptidoglycan synthesis. In consequence of the peptidoglycan biosynthesis inhibition, gene expression for cell wall lytic enzymes (*atl*, SA0423, SA2100) was negatively affected. In terms of membrane trafficking, the oligopeptide transport system genes *oppBCDF *and the components of the phosphotransferase system (PTS) were upregulated. While an increased expression of the PTS may boost the import of glucose to supply additional energy, the induction of OppBCDF might support cell wall biosynthesis by the acquisition of essential amino acids.

Since incubation with 0.5 × MIC of mersacidin was sufficient to massively induce the cell wall stress response, we further examined the gene expression profile of strain SA137/93G at even lower concentrations using DNA microarrays (Table 1). Incubation with 0.15 × MIC (4 μg/ml, 2.175 μM) of mersacidin was sufficient to significantly alter the expression of 19 out of 46 genes of the VraSR regulon [12] (Table 1) and 12 out of 17 genes belonging to the core cell wall stress stimulon [14]. These results show, that inhibitory antibiotic concentrations are not an obligatory requirement for the induction of the cell wall stress stimulon as it is the case for e.g. vancomycin and oxacillin [14,25,26]. Compared to 0.5 × MIC, the response was reduced after employing 0.15 × MIC in strain SA137/93G (Table 1), suggesting a dose-dependent effect on gene expression.

### Induction of *vraS *transcription by mersacidin

**Figure 2:**
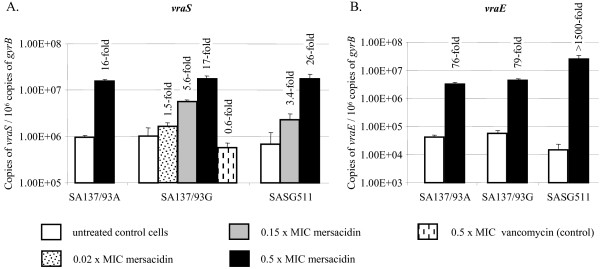
**Gene regulatory response of *S. aureus *strains SA137/93A, SA137/93G and SG511 to the lantibiotic mersacidin**. (A) qRT-PCR of *vraS *gene induction in response to subinhibitory concentrations of mersacidin and vancomycin (control). (B) qRT-PCR of *vraE *gene expression upon mersacidin treatment. The qRT-PCR values represent the mean of at least two independent experiments. Quantitative data are presented in relation to 10^6 ^copies of the housekeeping gene *gyrB*. Fold changes were calculated in relation to the untreated control cells, i. e. transcription levels in the absence of mersacidin.

To verify the induction of the cell wall stress response by the autoregulatory VraSR TCRS [27], overexpression of the sensor histidine kinase gene *vraS *was further analyzed via qRT-PCR (Fig. 2A). In the presence of 0.5 × MIC of mersacidin, a strong induction of *vraS *transcription (16- to 26-fold) was observed in all strains tested. Even 0.15 × MIC of mersacidin considerably induced *vraS *gene transcription in strain SA137/93G (5.6-fold) and strain SG511 (3.4-fold) (strain SA137/93A not tested). As a control, strain SA137/93G was incubated with subinhibitory concentrations of vancomycin (4 μg/ml, 2.75 μM, 0.5 × MIC). Here, no induction was visible by qRT-PCR. This is also confirmed by earlier studies of other groups who employed multiple MICs (8 to 10 × MICs) of cell wall affecting agents like vancomycin, oxacillin or bacitracin to induce the cell wall stress response [12-14], while low inhibitory or subinhibitory concentrations of vancomycin or oxacillin did not lead to an induction [14,25,26]. Likewise, an induction of the cell wall stress response could not be observed for the lantibiotic nisin at inhibitory concentrations [16]. Nisin acts by fast depolarization of the cell membrane as a cause of lipid II-based pore formation rather than by inhibition of cell wall biosynthesis [17,28]. Thus, the bacteria will be rapidly inactivated by nisin which prevents an adequate response to cell wall damage through the VraSR-stimulon. Hence, mersacidin turned out to be a strong inducer of the cell wall stress response in *S. aureus *compared to other substances like vancomycin, oxacillin, bacitracin and nisin.

A comparison of the transcript levels of *vraSR *of all tested strains showed that the levels did not vary significantly between the strains (Fig. 2A). Since the *vraSR *genes have been found to be more highly expressed in some VISA compared to VSSA, the VraSR TCRS has been proposed to be involved in the VISA-type resistance mechanism via contribution to cell wall thickening [14,29] and the deletion of the *vraSR *locus has been described to influence the development of resistance towards glycopeptides and β-lactams [12]. However, the cell wall stress response was similarly induced upon mersacidin treatment in the VISA strains as well as in the susceptible strain in our studies and all strains showed equal basal transcript levels. Interestingly, the induction of VraSR did not depend on the absolute concentration of mersacidin in the medium but seemed to be triggered by the stress itself, since 0.5 μg/ml of mersacidin massively induced the cell wall stress response in the susceptible strain SG511 (here 0.5 μg/ml correspond to 0.5 × MIC mersacidin, Fig. 2A), while *vraS *transcription levels were not significantly affected in strain SA137/93G (for this strain 0.5 μg/ml correspond to 0.02 × MIC mersacidin). Therefore, the VraSR TCRS might have evolved as a damage-sensing system that is indirectly induced as a cause of cell wall damage rather than a drug sensing system that recognizes the drug itself at defined concentrations. This is also indicated by findings showing that VraSR responds to various cell wall-active antibiotics as well as other conditions of cell wall stress. In this regard, the bacitracin-dependent induction of the LiaRS TCRS in *Bacillus subtilis*, a close homologue of VraSR, also indicated a damage-sensing mechanism [30]. This supports the idea of VraSR being a common accelerator system of peptidoglycan synthesis [12], which reacts to perturbations of cell wall integrity.

### Extensive upregulation of the *vraDE *ABC transporter genes and the effect of VraDE on the sensitivity towards mersacidin

The presence of mersacidin significantly influenced the regulation of several genes coding for hypothetical ABC transporters such as SA0192, *vraFG *and *vraDE *(see Additional file 1 and Additional file 2). Indeed, the most prominent event of gene regulation involved the upregulation of *vraDE *(40 to 70-fold in the microarray studies). The induction of *vraDE *gene transcription was further verified via qRT-PCR (Fig. 2B). Upon incubation with 0.5 × MIC, *vraDE *expression was induced 76 to 79-fold in strain SA137/93A and SA137/93G, respectively. In the susceptible strain SG511, the transcript level of the *vraDE *genes rose almost 1700-fold upon incubation with mersacidin. This transporter has been previously shown to be inducible by vancomycin and cationic AMPs (CAMPs) [20,29,31] and seems to be involved in the increased resistance towards bacitracin and the human β-defensin 3 (hBD3) in *S. aureus *[20]. However, the regulation underlying *vraDE *induction has not been identified yet. The VraSR TCRS does not seem to be solely involved in the upregulation of *vraDE *transcription as knock-out mutations of the respective gene locus did not lead to an altered expression of the *vraDE *genes [12]. The upregulation to such an extent might indicate that VraDE plays a critical role in the staphylococcal defense against mersacidin, especially since resistance is conferred by an ABC transporter in the producer strain of mersacidin [32].

To test whether VraDE is able to transport mersacidin out of the cell membrane and therefore supports a resistance phenotype, the growth behaviour and MICs of a *vraE *knock-out mutant of *S. aureus *SG511 [20] and its parent strain were examined in the presence of the lantibiotic. Since *S. aureus *SG511Δ*vraE *had displayed significantly reduced resistance to bacitracin and the lantibiotics nisin and Pep5 [20], VraDE was considered to be functional in the parent strain. Unexpectedly, *S. aureus *SG511Δ*vraE *did not show increased susceptibility towards mersacidin neither in growth curve recordings (data not shown) nor in MIC studies (MIC of SG511Δ*vraE*: 1 μg/ml; SG511: 1 μg/ml). Subsequent nucleotide sequencing of the *vraDE *genes including the promoter region revealed, that the -35 and the -10 region as well as the ribosomal binding site and the *vraD *gene were highly conserved in strains SA137/93A and SG511 (100% sequence identity) and that the *vraE *gene showed 95.7% sequence identity with mostly conservative amino acid substitutions (data not shown). Therefore, VraDE is unlikely to contribute to mersacidin resistance, presumably because it is unable to transport the lantibiotic. This could be due to the properties of mersacidin which is globular, neutral and non-membrane disturbing. Since VraDE has been shown to predominantly transport linear, cationic, membrane interacting compounds, it might be unable to facilitate the transport of other antimicrobials as also shown for chloramphenicol and oxacillin [20]. However, it cannot be ruled out that the knockout phenotype is indiscernible through the activity of another ABC transporter.

### Influence of a thickened cell wall of *S. aureus *on the activity of mersacidin

**Figure 3:**
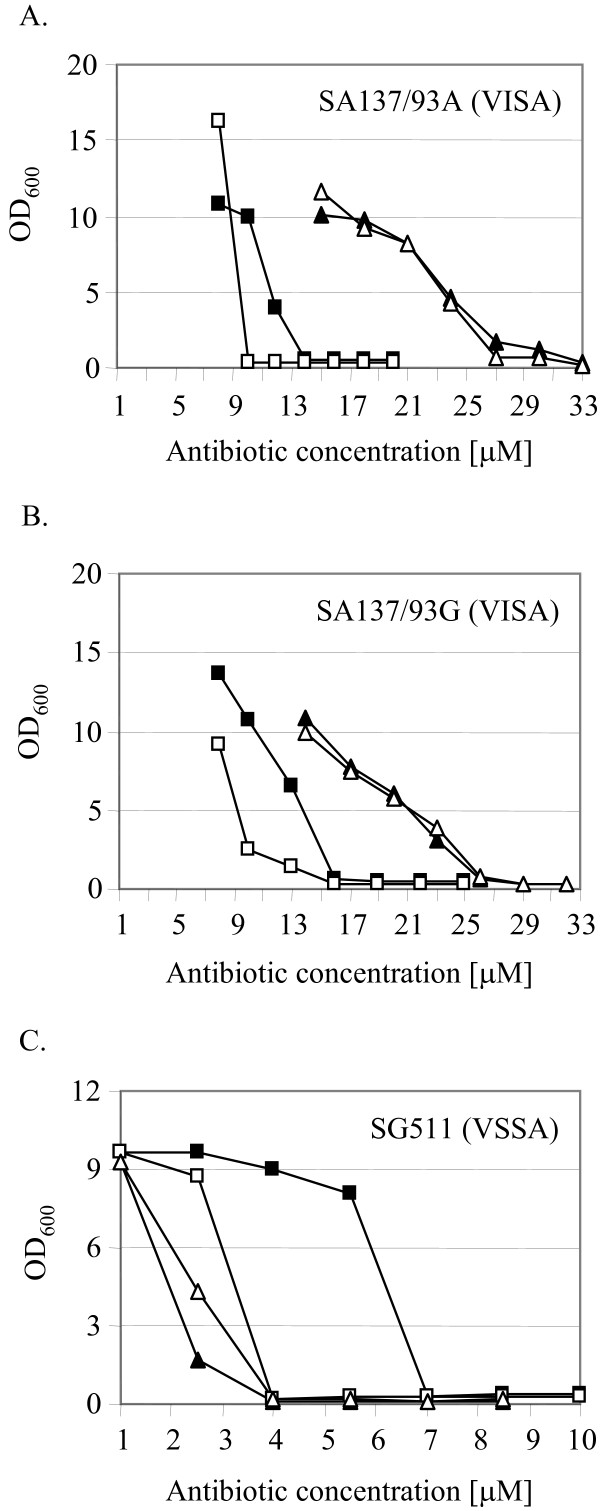
**Influence of cell wall thickness on mersacidin activity**. The influence of an increased cell wall thickness on the efficacy of mersacidin was studied for the *S. aureus *strains SA137/93A (A), SA137/93G (B) and SG511 (C). To this end, *S. aureus *cells were incubated in resting medium supplemented with glucose (RM+g), which allows the synthesis of increased amounts of peptidoglycan, or in the absence of glucose (RM-g), which prevents synthesis of extra cell wall material. The data represent the optical density (600 nm) of RM-preincubated *S. aureus *cells after treatment with different concentrations of mersacidin. Vancomycin served as a control. Black triangle, mersacidin/RM+g; white triangle, mersacidin/RM-g; black square, vancomycin/RM+g; white square, vancomycin/RM-g.

The VISA strains tested here are characterized by a cell wall with an increased thickness, which is also formed in the absence of antibiotics [18]. Actually, vancomycin intermediate resistance partly relies on an increase in cell wall material [18,21,22], which might result from an activated cell wall metabolism. In order to estimate the influence of an increased cell wall thickness on the action of mersacidin, cells with increased amounts of peptidoglycan were obtained by incubation in resting medium (RM) for two hours. Cell wall resting medium allows the biosynthesis of excess cell wall material, while the absence of essential amino acids prohibits growth. Controls were performed in RM devoid of glucose (RM-g), which is necessary for synthesis of extra cell wall material [21,33]. After incubation of the cells in RM or RM-g, the susceptibility to mersacidin was tested. Vancomycin served as control in our studies. In the case of mersacidin, the incubation in the presence of glucose did not decrease the susceptibility of *S. aureus *SG511 and of the VISA strains SA137/93A and SA137/93G (Fig. 3), whereas the efficacy of vancomycin was always lower against cells that had been incubated in the presence of glucose. In strain SG511, mersacidin was even more effective than vancomycin, which was not the case for the strains SA137/93A and SA137/93G.

The above results demonstrate that the cell wall thickness does not impact on the antibiotic efficacy of mersacidin. Thus, the induction of the cell wall stress response at low mersacidin concentrations may be partly explained by the differences that characterize the interactions of mersacidin and vancomycin with the cell envelope. While mersacidin and vancomycin both target lipid II, vancomycin acts by binding to the D-alanyl-D-alanine terminus of the peptide side chain of lipid II. In a thickened cell wall, increased amounts of free D-alanyl-D-alanine termini provide false target sites for vancomycin binding. This results in a decreased diffusion velocity of vancomycin through the cell wall, the "clogging effect" [21], and only those vancomycin molecules that are not trapped in the cell wall will reach lipid II. In contrast to vancomycin, mersacidin complexes the sugar phosphate head group of the peptidoglycan precursor lipid II, which is only present in the membrane and experiments with radiolabeled mersacidin have confirmed that it does not bind to isolated cell walls [7]. In *in vitro *cell wall biosynthesis systems, the effective concentration of mersacidin is in the same range as that of vancomycin [34]. Therefore, although nearly similar molar concentrations (2.175 μM mersacidin (0.15 × MIC) and 2.75 μM vancomycin (0.5 × MIC)) of the two antibiotics were used in the induction assays, less vancomycin than mersacidin molecules may have been available at the cell membrane for the binding of lipid II. This effect could explain why higher concentrations of vancomycin have to be employed in order to induce the cell wall stress response and why lower initial concentrations of mersacidin may be sufficient for an effective induction of VraSR.

## Conclusion

Mersacidin strongly induces the cell wall stress response of *S. aureus *even at very low concentrations as compared to other antibiotics like nisin, vancomycin, bacitracin or oxacillin, thereby reflecting its unique mode of action. Our results underline the important role of lipid II in this context and demonstrate that the induction of the cell wall stress response is not in every case dependent on inhibitory concentrations of cell wall-active substances. Furthermore, mersacidin represents a molecule that seems not to be a substrate for the multidrug resistance transporter VraDE which apparently functions in the first line bacterial counter strategy against bacitracin and cationic toxic compounds. Biochemical characterization of VraDE and related transporters will test this hypothesis and may provide directions for circumventing the activity of resistance transporters through structural modification of antibiotics.

## Methods

### Bacterial strains, growth conditions and antimicrobial susceptibility testing

Bacterial strains and plasmids are listed in Table 2. *S. aureus *strains were cultured in brain heart infusion (BHI) medium (Becton-Dickinson GmbH, Heidelberg, Germany) at 37°C with aeration unless indicated otherwise. MIC determinations of mersacidin (Hoechst, Frankfurt am Main, Germany) were performed in polystyrene round bottom microtiter plates (Greiner, Frickenhausen, Germany) using BHI broth. An inoculum of 5 × 10^5 ^CFU/ml was employed in the arithmetic broth microdilution method. All experiments were done in triplicate. For growth analysis and RNA preparations, *S. aureus *cultures were diluted 200-fold from overnight cultures and grown to exponential phase until they reached an OD_600 _of 0.5 in BHI broth. Then, mersacidin was added as indicated, and the cultures were further grown to an OD_600 _of 1.0 for RNA preparations (~30 min) or longer to assess growth behavior. CaCl_2 _was supplemented to all cultures to a final concentration of 1 mM, since Ca^2+^-ions enhance the bactericidal effect of mersacidin [35,36].

**Table 2:** Bacterial strains, plasmids and primers used in this study

Strain, plasmid or primer	Relevant characteristic(s)/primer sequence	Reference(s) or source
Strains		
*S. aureus*		
SG511	Susceptible control strain	RKI Berlin, Germany
SA137/93A	Clinical hVISA isolate; MET^r^, Northern German epidemic MRSA	[18]
SA137/93G	Spontaneous mutant of SA137/93A, ΔSCC *mec *(MET^s^), Δ*tcaA*	[18,19]
SG511Δ*vraE*	*vraE*-defective mutant of strain SG511	[20]
		
Plasmids		
pUC19*gyrB*	pUC19 (Amp^r^), carrying a 560 bp internal fragment of *gyrB*; external plasmid standard for qRT-PCR	[38]
		
Primers		
a) Oligonucleotide primers used for the synthesis of external qRT-PCR standards		
vraE-1	TCTCATATGACATTTAACCATATCGTTTTC	This study
vraE-2	TAACTCGAGAATGGTTTTCTTAATCAATTTGTTTG	This study
vraS-1	TTACATATGAACCACTACATTAGAACAAT	This study
vraS-2	AATAAGCTTATCGTCATACGAATCCTCCT	This study
b) Oligonucleotide primers used for qRT-PCR		
gyrB-297	TTAGTGTGGGAAATTGTCGATAAT	[39]
gyrB-547	AGTCTTGTGACAATGCGTTTACA	[39]
vraE-1-RT2	GTAACTGTATTGTGTTTCGCGGC	This study
vraE-2-RT2	TGATGGCATTGTTGCCTGTTACC	This study
vraS-1-RT	GTTGGTTCGGTACTCGCATA	This study
vraS-2-RT	CTCGAGCTAGTCTTTGACGTTC	This study

Abbreviations: MET, methicillin; Amp, ampicillin; r, resistant; s, susceptible

### Preparation of total RNA

Exponential-phase cultures of 10 ml were grown as aforementioned and stabilized by incubation with two volumes of prewarmed RNAprotect Bacteria Reagent (Qiagen, Hilden, Germany) for 5 min at 37°C. The culture was subsequently harvested by centrifugation and the pellets were shock-frozen in liquid nitrogen and kept at -70°C. The cells were lysed in the presence of 400 μg/ml lysostaphin (Genmedics, Reutlingen, Germany) and total RNA was extracted using the PrestoSpin R bug kit including DNase I treatment (Molzym, Bremen, Germany) following the manufacturer's instructions. Quality and quantity of total RNA were determined by agarose gel electrophoresis and measured by using the Nanodrop spectrophotometer (Nanodrop Technologies, Wilmington, USA).

### Synthesis of CyDye-3 and CyDye-5 labeled cDNA for microarray experiments

Fluorescence-labeled single-stranded cDNA was obtained by reverse transcription of total RNA. To this end, aliquots of total RNA preparations from three different cultures of the respective experiment were pooled to a total amount of 9 μg (3 μg each) and transcribed into cDNA using 100 units (U) of BioScript reverse transcriptase (Bioline, Luckenwalde, Germany) following the manufacturer's instructions. For direct cDNA labeling, the total reaction volume of 40 μl contained 75 μg/ml pd(N)6 random hexamers (GE Healthcare – Amersham, NJ, USA), 0.1 mM CyDye3- or CyDye5-dCTPs (GE Healthcare – Amersham) aside from 0.2 mM dCTP, 0.5 mM dATP, 0.5 mM dTTP, 0.5 mM dGTP and 25 U/ml RNase-OUT (Invitrogen, Karlsruhe, Germany). RNA was degraded by alkaline hydrolysis at 65°C and fluorescence-labeled cDNA was purified using the MinElute PCR purification kit (Qiagen). cDNA synthesis and CyDye3/CyDye5 incorporation were verified by using the Nanodrop spectrophotometer (Nanodrop Technologies).

### Microarray hybridization and analysis

Microarray-based transcriptional profiling by competitive hybridization of fluorescence-labeled cDNA was performed by using the custom PCR product full-genome chip *sciTracer *(Scienion, Berlin, Germany). Each experiment was performed 4 times including a dye swap resulting in four chips per competitive comparison to increase reproducibility. Only strain SA137/93G incubated with 0.15 × MIC of mersacidin was reproduced in duplicate. All hybridizations were done with equal amounts of cDNA probes displaying similar picomoles of incorporated dye. Fluorescence-labeled cDNA probes were mixed in hybridization buffer (Scienion) in a total volume of 55 μl, denatured at 95°C for 2 min and subsequently applied to the microarray slide followed by incubation at 42°C for 72 hours under humidified conditions according to the manufacturer's instructions. Hybridized microarrays were washed at room temperature in SSC buffer with decreasing salt concentrations (1 × SSC/0.3% SDS for 5 min, 0.2 × SSC for 5 min, 0.06 × SSC for 30s). For image capture, the microarray was scanned with a GenePix 4000B scanner (Axon Instruments/Distribution by Biozyme Scientific GmbH, Hessisch Oldendorf, Germany). The TIFF images were analyzed with GenePixPro4.1 software (Axon Instruments). The actual signal intensity was calculated by using the mean of the "median of ratios" of the individual spot. The data sets were then normalized by using Acuity 3.1 software (Axon Instruments) and by applying the LOWESS algorithm. Significant changes of gene expression were determined by implementing SAM (significance analysis of microarrays; ; [37]) using the one class response and a false discovery rate of < 1% with a medium number of falsely called significant genes of < 1.

### Microarray data accession number

Additional information on the microarray platform as well as the processed and raw microarray data of this study have been deposited in NCBI's Gene Expression Omnibus (GEO)  to be found under the GEO Series accession number GSE9261.

### Microarray validation and transcript quantification by Real-Time PCR

The LightCycler instrument (Roche Diagnostics, Mannheim, Germany) was employed to generate quantitative transcription data by measuring sample amplification during the log-linear phase of the PCR. Therefore, total RNA preparations (3 μg) were transcribed into cDNA using BioScript reverse transcriptase (Bioline) and pd(N)6 random hexamers (GE Healthcare) following the manufacturers' instructions. Quantitative Real-Time PCR (qRT-PCR) was performed by using the LightCycler FastStart DNA Master SYBR Green I kit (Roche Diagnostics) according to the manufacturer's instructions. For all experiments, the amount of transcripts was determined from the appropriate standard curve and the target concentration was expressed in relation to the concentration of the constitutively expressed housekeeping gene *gyrB*. Each standard curve was generated by assaying gene specific PCR product or plasmid templates. The specific primers, which were used for the synthesis of external LightCycler standards or for qRT-PCR of the target genes and the endogenous control *gyrB*, are listed in Table 2. To control quality and reproducibility of the qRT-PCR data, at least two different cDNA probes were synthesized employing RNA preparations from independent cultures for every condition. The PCR products were verified by melting curve analysis and ethidium bromide staining on agarose gels.

### Growth experiments after incubation in resting medium

To obtain cells with different amounts of peptidoglycan, *S. aureus *cultures were grown to an OD_600 _of 0.5 in 35 ml BHI, harvested, washed and resuspended in 35 ml prewarmed resting medium (RM). RM contains salts, amino acids (glutamic acid 0.3 g/l, aspartic acid 0.1 g/l, lysine 0.1 g/l, alanine 0.2 g/l, cystine 0.2 g/l) and glucose (10 g/l) necessary for cell wall biosynthesis but not sufficient for growth and division. As a control, the omission of glucose (RM-g) also stopped cell wall biosynthesis [21,33]. After 2 h of incubation at 37°C, the cells were harvested again, resuspended in 30 ml fresh BHI broth and aliquots of 3 ml were distributed into tubes containing different concentrations of mersacidin or vancomycin (control). The tubes were incubated overnight on a shaker and after 20 h the optical density (600 nm) of the cultures was measured.

## Authors' contributions

PS carried out the microarray and qRT-PCR studies, tested the strains and wrote the manuscript. AJ participated in the microarray studies and CS performed the growth experiments in cell wall resting medium. VS and HGS constructed the *vraDE *knockout clone. GB conceived the study, participated in its design and corrected the manuscript. All authors read and approved the final manuscript.

## Supplementary Material

Additional file 1 **Table A1 Upregulated genes**. Genes with significantly increased expression of at least 2.5-fold in this study. Click here for file

Additional file 2 **Table A2 Downregulated genes**. Genes with significantly decreased expression of at least 2.5-fold in this study. Click here for file
