# Supplementary material for: The lantibiotic mersacidin is a strong inducer of the cell wall stress response of Staphylococcus aureus
Source: BMC Microbiol. 2008 Oct 23;8:186. doi: 10.1186/1471-2180-8-186 (PMC2592248; doi:10.1186/1471-2180-8-186)
Supplement: Additional file 1 — Table A1 Upregulated genes. Genes with significantly increased expression of at least 2.5-fold in this study. [file 1471-2180-8-186-S1.pdf]

TABLE A1. Genes with significantly increased expression of at least 2.5-fold in this study[1]

N315 ORF	Gene	Gene product	Fold change[2]		
			SA137/93A (0.5xMIC)	SA137/93G (0.5xMIC)	SG511 (1xMIC)
Adaption to atypical conditions					
SA0483	*clpC*	endopeptidase	1.2	**2.7**	**6.6**
SA0659		hypothetical protein, similar to CsbB stress response protein	2.1	**3.5**	3.0
SA0723	*clpP*	ATP-dependent Clp protease proteolytic subunit homologue	1.3	**3.0**	**3.4**
SA0755		hypothetical protein, similar to general stress protein 170	1.7	**4.0**	**2.8**
SA0835	*clpB*	ClpB chaperone homologue	1.5	6.9	**16.4**
SA1146	*bsaA*	glutathione peroxidase	2.1	**2.7**	2.7
SA1410	*grpE*	GrpE protein	0.9	3.1	**5.3**
SA1549		hypothetical protein, similar to serine proteinase Do, heat-shock protein htrA	**7.1**	**4.6**	**5.6**
SA2175		hypothetical protein, similar to small heat shock protein	2.4	**3.0**	3.1
SA2336	*clpL*	ATP-dependent Clp proteinase chain clpL	0.9	**4.0**	1.3
Cell division					
SA1023	ftsL	cell division protein	**2.4**	1.6	**3.0**
Cell wall related genes					
SA0205		hypothetical protein, similar to lysostaphin precursor	**6.0**	**4.5**	**13.6**
SA0244		hypothetical protein, similar to teichoic acid biosynthesis protein F	1.6	1.4	**2.8**
SA0265	*lytM*	peptidoglycan hydrolase	**4.0**	**2.1**	1.7
SA0638	*bacA*	bacitracin resistance protein (putative undecaprenol kinase) homologue	**3.0**	**1.9**	1.8
SA1103	*uppS*	undecaprenyl pyrophosphate synthetase	**2.5**	**3.0**	2.0
SA1266		hypothetical protein, similar to cell wall enzyme EbsB	3.4	1.9	**3.7**
SA1283	*pbp2*	penicillin binding protein 2	3.4	2.3	**3.5**
SA1691	*sgtB*	hypothetical protein, similar to penicillin-binding protein 1A/1B	**13.1**	**8.8**	**9.7**
SA1926	*murZ*	UDP-N-acetylglucosamine 1-carboxylvinyl transferase 2	3.2	**4.4**	**6.1**
SA2481		conserved hypothetical protein	**2.4**	**3.5**	**7.0**
Detoxification					
SA0128	*sodM*	superoxide dismutase	1.0	X	1.0
SA0681		hypothetical protein, similar to multidrug resistance protein	1.2	**3.1**	1.0
SA1170	*katA*	Catalase	1.6	**2.7**	1.9
SA1238		hypothetical protein, similar to tellurite resistance protein	**2.6**	**3.2**	**3.3**
SA1382	*sodA*	superoxide dismutase SodA	1.1	**3.0**	1.8
DNA replication, modification, recombination and repair					
SA1282	*recU*	recombination protein U homologue	**3.6**	2.5	**2.5**
SA1313		probable ATP-dependent DNA helicase RecQ	2.0	**2.2**	**2.7**
SA1485	*radC*	truncated hypothetical protein, similar to DNA repair protein	1.5	1.4	**6.3**
SA1711		hypothetical protein, similar to DNA-damage inducible protein P	2.2	**1.6**	**3.4**
SA2335	*adaB*	probable methylated DNA-protein cysteine methyltransferase	1.7	**2.8**	2.2
Membrane bioenergetics (electron transport chain and ATP synthase)					
SA0211		hypothetical protein, similar to NADH-dependent dehydrogenase	2.2	1.3	**4.0**
SA0411	*ndhF*	NADH dehydrogenase subunit 5	1.6	1.0	**2.9**
SA0817		hypothetical protein, similar to NADH-dependent flavin oxidoreductase	2.1	**3.4**	**2.4**
SA1221		thioredoxine reductase	1.2	**2.6**	1.2
SA2324		hypothetical protein, similar to thioredoxin	1.3	**2.6**	**3.0**
Metabolism of amino acids					
SA0011		hypothetical protein, similar to homoserine-o-acetyltransferase	**1.6**	**2.0**	**2.8**
SA0430	*gltB*	glutamate synthase large subunit	**2.8**	**2.0**	1.3
SA0471	*cysK*	cysteine synthase (o-acetylserine sulfhydrylase) homologue	1.6	**3.4**	1.8
SA0512	*ilvE*	branched-chain amino acid aminotransferase homologue	2.1	**2.5**	2.1
SA0829		hypothetical protein, similar to 5-oxo-1,2,5-tricarboxilic-3-penten acid decarboxylase	2.1	**3.2**	**3.1**
SA0902		HisC homologue	2.2	1.8	**4.2**
SA0942	*pdf1*	formylmethionine deformylase homologue	1.7	1.6	**2.9**
SA1164	*dhoM*	homoserine dehydrogenase	**2.5**	**2.7**	**6.2**
SA1165	*thrC*	threonine synthase	1.6	2.0	**3.9**

continued on following page

TABLE A1 - *Continued*

N315 ORF	Gene	Gene product	Fold change[2]		
			SA137/93A (0.5xMIC)	SA137/93G (0.5xMIC)	SG511 (1xMIC)
SA1166	*thrB*	homoserine kinase homologue	1.4	1.7	**3.9**
SA1216		hypothetical protein, similar to oligoendopeptidase	1.8	**1.7**	**2.8**
SA1226	*asd*	aspartate semialdehyde dehydrogenase	2.2	**3.8**	2.2
SA1227	*dapA*	dihydrodipicolinate synthase	**2.3**	**3.0**	2.0
SA1228	*dapB*	dihydrodipicolinate reductase	**2.3**	**2.6**	1.9
SA1232	*lysA*	diaminopimelate decarboxylase	**2.1**	**2.4**	**2.7**
SA1531	*ald*	alanine dehydrogenase	2.1	**1.5**	**4.2**
SA1545	*serA*	D-3-phosphoglycerate dehydrogenase	2.1	**1.6**	**2.8**
SA1861	*ilvC*	alpha-keto-beta-hydroxylacil reductoisomerase	**1.9**	**2.5**	1.2
SA2341	*rocA*	1-pyrroline-5-carboxylate dehydrogenase	1.2	1.4	**3.1**
Metabolism of carbohydrates					
SA0528		hypothetical protein, similar to hexulose-6-phosphate synthase	1.5	**3.4**	3.3
SA0658		hypothetical protein, similar to plant-metabolite dehydrogenases	1.5	**3.3**	**2.6**
SA0958		myo-inositol-1(or 4)-monophosphatase homologue	1.8	**3.1**	**3.1**
SA1142	*glpD*	aerobic glycerol-3-phosphate dehydrogenase	1.4	**2.0**	**3.8**
SA1566		endo-1,4-beta-glucanase homologue	**2.0**	**2.9**	2.4
SA1599		hypothetical protein, similar to transaldolase	1.7	**2.7**	**3.1**
SA1736	*aldH*	aldehyde dehydrogenase	2.0	**2.3**	**3.3**
SA1924		hypothetical protein, similar to aldehyde dehydrogenase	1.3	**3.4**	2.4
SA1925		conserved hypothetical protein	**3.5**	**3.8**	**4.7**
SA2104		hypothetical protein, similar to suppressor protein SuhB	1.4	**2.0**	**2.9**
SA2266		hypothetical protein, similar to oxidoreductase	**2.0**	**3.4**	1.6
SA2346		hypothetical protein, similar to D-specific D-2-hydroxyacid dehydrogenase ddh homologue	1.9	**2.2**	**3.8**
SA2490		hypothetical protein, similar to N-hydroxyarylamine O-acetyltransferase	**3.6**	**6.0**	**5.4**
Metabolism of coenzymes and prosthetic groups					
SA0915	*folD*	FolD bifunctional protein	1.3	**2.7**	1.8
SA2412		hypothetical protein, similar to uroporphyrin-III C-methyltransferase	1.9	**2.3**	**2.7**
SA2438		hypothetical protein, similar to N-carbamoylsarcosine amidohydrolase	**2.3**	**2.7**	**3.7**
Metabolism of lipids					
SA0534	*vraB*	acetyl-CoA c-acetyltransferase	**18.5**	**8.5**	**12.9**
SA0572		hypothetical protein, similar to esterase/lipase	0.9	**3.3**	1.8
SA1435		hypothetical protein, similar to acetyl-CoA carboxylase (biotin carboxyl carrier subunit), *accB* homologue	0.9	1.3	**3.4**
SA1542		hypothetical protein, similar to glycerophosphoryl diester phosphodiesterase	2.3	**2.8**	**4.6**
SA2240		hypothetical protein, similar to para-nitrobenzyl esterase chain A	1.8	**4.1**	2.2
Metabolism of nucleotides and nucleic acids					
SA0864		GTP pyrophosphokinase	1.6	1.8	**2.8**
SA1172		hypothetical protein, similar to GMP reductase	1.8	**3.0**	2.4
SA1308		30S ribosomal protein S1	2.0	**3.5**	**3.2**
SA2297		hypothetical protein, similar to GTP-pyrophosphokinase	**9.1**	**14.1**	**10.3**
Metabolism of phosphate					
SA1237		hypothetical protein, similar to 5-bromo-4-chloroindolyl phosphate hydrolysis protein XpaC	**2.5**	**4.1**	**6.0**
SA2301		hypothetical protein, similar to alkaline phosphatase	2.7	**2.7**	3.3
Pathogenic factors (toxins and colonization factors)					
SA0382	*set6*	exotoxin 6 [Pathogenicity island SaPIn2]	2.4	**2.7**	**5.1**
SA0610		hypothetical protein, similar to lipase LipA	1.2	**0.7**	**3.9**
SA0909	*fmtA*	FmtA, autolysis and methicillin resistant-related protein	**9.3**	3.8	**6.3**
SA2006		hypothetical protein, similar to MHC class II analogue	**3.4**	**7.3**	**4.4**
SA2323		conserved hypothetical protein	1.9	**2.4**	**2.9**
Phage-related functions					
SA1760		holin homologue [Bacteriophage phiN315]	**3.8**	3.2	**17.5**

continued on following page

TABLE A1 - *Continued*

N315 ORF	Gene	Gene product	Fold change[2]		
			SA137/93A (0.5xMIC)	SA137/93G (0.5xMIC)	SG511 (1xMIC)
Protein modification, folding and secretion					
SA0825	*spsA*	type-I signal peptidase	**2.2**	1.4	**3.9**
SA0826	*spsB*	type-1 signal peptidase 1B	**2.4**	1.5	**2.9**
SA1257		peptide methionine sulfoxide reductase MsrA2	**6.0**	**11.0**	**17.1**
SA1530		Xaa-Pro dipeptidase homologue	1.8	**3.1**	2.2
SA1409	*dnaK*	DnaK protein	0.9	**2.1**	**4.6**
SA1659	*prsA*	peptidyl-prolyl cis/trans isomerase homologue	**10.8**	**18.1**	**33.3**
SA1836	*groEL*	GroEL protein	1.2	**3.9**	4.5
Protein transport and binding					
SA0166		hypothetical protein, similar to nitrate transporter	2.0	**2.4**	**2.6**
SA0531	*proP*	proline/betaine transporter homologue	2.4	**2.7**	0.6
SA0640		hypothetical protein, similar to ABC transporter required for expression of cytochrome bd	1.6	1.5	**2.6**
SA0677		hypothetical protein, similar to choline transport ATP-binding protein	1.2	1.1	**2.8**
SA0769		ABC transporter ATP-binding protein homologue	0.9	1.4	**3.6**
SA0845	*oppB*	oligopeptide transport system permease protein	1.4	**3.6**	**3.5**
SA0846		hypothetical protein, similar to oligopeptide transport system permease protein OppC	**1.8**	**3.1**	**3.4**
SA0847	*oppD*	oligopeptide transport system ATP-binding protein OppD homologue	1.5	**3.0**	**2.6**
SA0848	*oppF*	oligopeptide transport system ATP-binding protein OppF homologue	2.3	**3.9**	2.1
SA1255		PTS system, glucose-specific enzyme IIA component	**4.7**	**10.7**	**13.1**
SA1478		hypothetical protein, similar to transporter PAB2175 from *Pyrococcus abyssi*	**3.8**	**3.7**	**4.9**
SA1654		hypothetical protein, similar to ABC transporter EcsB	2.3	**2.0**	**2.9**
SA1655		ABC transporter EcsA homologue	1.5	**2.4**	**5.1**
SA1848	*nrgA*	probabale ammonium transporter	1.4	1.4	**4.1**
SA1948	*czrB*	cation-efflux system membrane protein homologue	0.9	**1.9**	**2.7**
SA2112		hypothetical protein, similar to sodium-dependent transporter	1.9	1.3	**2.9**
SA2114	*glvC*	PTS system, arbutin-like IIBC component	**3.7**	**5.6**	**4.7**
SA2148		hypothetical protein, similar to membrane protein	1.4	1.6	**4.1**
SA2396		hypothetical protein, similar to amino acid transporter	1.9	**1.3**	**3.0**
SA2416		hypothetical protein, similar to ABC transporter (ATP-binding protein)	**2.3**	**2.3**	**2.7**
SA2492	*vraD*	hypothetical protein, similar to ABC transporter	**59.4**	**72.2**	**52.7**
SA2493	*vraE*	hypothetical protein, similar to ABC transporter (permease)	**52.7**	**39.6**	**42.4**
RNA synthesis - regulation					
SA0480	*ctsR*	transcription repressor of class III stress genes homologue	1.1	**4.2**	**5.0**
SA1195	*msrR*	peptide methionine sulfoxide reductase regulator MsrR	**4.2**	**3.6**	**5.5**
SA1411	*hrcA*	Heat-inducible transcriptional repressor	1.3	**3.3**	**5.1**
SA1700	*vraR*	two-component response regulator	**13.3**	**8.0**	**17.3**
SA1947	*czrA*	repressor protein	1.2	**2.5**	**3.5**
SA2103		hypothetical protein, similar to lyt divergon expression attenuator LytR	**6.4**	**6.1**	**8.0**
SA2159		hypothetical protein, similar to transcription repressor of sporulation, septation and degradation PaiA	1.8	**2.1**	**3.4**
SA2296		hypothetical protein, similar to transcriptional regulator, MerR family	**8.1**	**10.4**	**5.5**
SA2458	*icaR*	*ica* operon transcriptional regulator IcaR	1.4	**2.6**	1.6
Sensors (signal transduction)					
SA1701	*vraS*	two-component sensor histidine kinase	**11.7**	**8.9**	**17.0**
Transformation competence					
SA0857		hypothetical protein, similar to negative regulator of genetic competence MecA	3.0	3.4	**7.0**
Hypothetical proteins					
SA0165		hypothetical protein, similar to alpha-helical coiled-coil protein SrpF	1.8	**3.4**	**3.5**
SA0192		hypothetical protein, similar to ABC transporter ATP-binding protein	**23.6**	**31.7**	**26.6**
SA0358		conserved hypothetical protein	**10.6**	**15.4**	**39.3**
SA0359		conserved hypothetical protein	1.9	**6.1**	**4.3**

continued on following page

TABLE A1 - *Continued*

N315 ORF	Gene	Gene product	Fold change[2]		
			SA137/93A (0.5xMIC)	SA137/93G (0.5xMIC)	SG511 (1xMIC)
SA0481		conserved hypothetical protein	1.8	**5.3**	**8.0**
SA0482		hypothetical protein, similar to creatine kinase	1.2	**4.5**	**5.8**
SA0509		conserved hypothetical protein	1.1	**2.8**	0.8
SA0529		conserved hypothetical protein	1.4	**3.0**	**3.7**
SA0530		hypothetical protein, similar to indigoidine synthesis protein	**2.5**	**2.4**	1.3
SA0535	*vraC*	hypothetical protein	**25.7**	**11.3**	**18.9**
SA0536		hypothetical protein	**30.2**	**17.1**	**26.6**
SA0591		hypothetical protein	**11.3**	**8.9**	**10.9**
SA0608		hypothetical protein	**2.7**	**3.8**	**9.2**
SA0611		hypothetical protein	1.2	**0.8**	**4.6**
SA0612		conserved hypothetical protein	2.0	**2.5**	2.4
SA0637		conserved hypothetical protein	2.5	**4.0**	1.5
SA0647		hypothetical protein	2.3	1.2	**3.6**
SA0648		conserved hypothetical protein	**2.4**	1.5	**3.6**
SA0707		conserved hypothetical protein	**4.8**	**9.7**	2.5
SA0721		conserved hypothetical protein	1.0	**3.1**	1.9
SA0725		conserved hypothetical protein	2.9	**2.9**	1.8
SA0750		conserved hypothetical protein	1.9	1.7	**4.9**
SA0770		conserved hypothetical protein	0.8	1.2	**3.8**
SA0771		conserved hypothetical protein	1.2	1.4	**2.8**
SA0772		conserved hypothetical protein	2.4	**4.5**	0.8
SA0782		conserved hypothetical protein	2.1	1.6	**2.9**
SA0824		conserved hypothetical protein	**4.1**	**2.4**	**3.9**
SA0833		conserved hypothetical protein	1.8	**2.6**	1.8
SA0856		conserved hypothetical protein	2.0	**2.6**	2.1
SA0903		conserved hypothetical protein	3.2	1.4	**5.3**
SA0908		conserved hypothetical protein	2.2	1.5	**3.1**
SA0914		hypothetical protein, similar to chitinase B	**14.2**	**3.9**	**15.3**
SA0931		hypothetical protein	**4.9**	**3.2**	**6.2**
SA0943		conserved hypothetical protein	**3.2**	**3.2**	**4.5**
SA0957		conserved hypothetical protein	1.8	2.2	**3.2**
SA0962		conserved hypothetical protein	1.8	1.4	**2.8**
SA1018		conserved hypothetical protein	1.7	**2.2**	**3.0**
SA1019		conserved hypothetical protein	**3.9**	**6.9**	**6.5**
SA1022		conserved hypothetical protein	2.2	2.1	**2.8**
SA1050		conserved hypothetical protein	2.0	**2.6**	1.1
SA1173		conserved hypothetical protein	**2.7**	**2.8**	**3.7**
SA1185		conserved hypothetical protein	2.0	**1.9**	**3.2**
SA1186		conserved hypothetical protein	1.9	1.4	**3.4**
SA1256		conserved hypothetical protein	**7.9**	**16.1**	**18.8**
SA1280		conserved hypothetical protein	1.7	**2.8**	**2.8**
SA1281		conserved hypothetical protein	**1.9**	**3.0**	**2.9**
SA1293		conserved hypothetical protein	1.7	**2.3**	**3.5**
SA1419		conserved hypothetical protein	1.3	1.4	**4.6**
SA1436		conserved hypothetical protein	0.7	1.0	**3.4**
SA1476		hypothetical protein	**11.5**	**11.3**	**15.9**
SA1532		conserved hypothetical protein	2.5	**9.1**	**5.5**
SA1543		conserved hypothetical protein	2.8	**3.9**	**6.0**
SA1544		hypothetical protein, similar to soluble hydrogenase 42 kD subunit	1.8	**2.0**	**4.9**
SA1546		hypothetical protein	**2.3**	**1.7**	**3.2**
SA1606		plant metabolite dehydrogenase homologue	1.9	**3.5**	**2.6**
SA1649		conserved hypothetical protein	1.9	1.1	**3.0**

continued on following page

TABLE A1 - *Continued*

N315 ORF	Gene	Gene product	Fold change[2]		
			SA137/93A (0.5xMIC)	SA137/93G (0.5xMIC)	SG511 (1xMIC)
SA1680		conserved hypothetical protein	**2.3**	**2.7**	**3.0**
SA1682		conserved hypothetical protein	2.4	**2.9**	1.8
SA1686		conserved hypothetical protein	2.1	**2.1**	**3.5**
SA1690		conserved hypothetical protein	**2.0**	**2.1**	**4.0**
SA1692		conserved hypothetical protein	1.3	**3.1**	0.8
SA1702		conserved hypothetical protein	**10.4**	**9.4**	**12.2**
SA1703		hypothetical protein	**31.6**	**24.8**	**45.4**
SA1712		conserved hypothetical protein	**24.7**	**21.0**	**29.4**
SA1743		hypothetical protein	2.7	**4.3**	2.6
SA1942		conserved hypothetical protein	1.7	**3.7**	2.5
SA1990		conserved hypothetical protein	1.9	**2.6**	**3.9**
SA2004		conserved hypothetical protein	2.1	2.2	**2.5**
SA2049		hypothetical protein	4.3	**5.8**	6.5
SA2113		hypothetical protein	**14.9**	**11.8**	**11.4**
SA2138		conserved hypothetical protein	3.1	**4.2**	5.3
SA2139		hypothetical protein	2.7	**2.8**	3.4
SA2146	*tcaA*	TcaA protein	**3.3**	1.6	**4.0**
SA2158		hypothetical protein, similar to TpgX protein	2.1	**2.7**	**2.3**
SA2220		conserved hypothetical protein	**7.0**	**12.2**	**21.4**
SA2221		hypothetical protein	**25.6**	**18.3**	**42.7**
SA2366		conserved hypothetical protein	0.9	**2.7**	1.1
SA2367		conserevd hypothetical protein	1.0	**3.3**	1.1
SA2480	*drp35*	drug responsive protein 35	2.7	**3.4**	**2.8**

[1] Significant changes of gene expression were determined by implementing SAM (significance analysis of microarrays; http://www-stat.stanford.edu/~tibs/SAM/).
[2] Fold change in transcript level indicated as mean of the “median of ratios” compared to control cells. Fold change in bold = classified as "significantly" regulated in this strain by SAM. “X” indicates the deletion of this ORF in the respective strain according to Reipert et al. (2003).
