# Supplementary material for: The lantibiotic mersacidin is a strong inducer of the cell wall stress response of Staphylococcus aureus
Source: BMC Microbiol. 2008 Oct 23;8:186. doi: 10.1186/1471-2180-8-186 (PMC2592248; doi:10.1186/1471-2180-8-186)
Supplement: Additional file 2 — Table A2 Downregulated genes. Genes with significantly decreased expression of at least 2.5-fold in this study. [file 1471-2180-8-186-S2.pdf]

TABLE A2. Genes with significantly decreased expression of at least 2.5-fold in this study[1]

N315 ORF	Gene	Gene product	Fold change[2]		
			SA137/93A (0.5xMIC)	SA137/93G (0.5xMIC)	SG511 (1xMIC)
Adaption to atypical conditions					
SA0747	*cspC*	cold-shock protein C	0.65	0.84	**0.40**
SA1984	*asp23*	alkaline shock protein 23, ASP23	0.64	2.24	**0.32**
Cell division					
SA0905	*atl*	autolysin	**0.42**	0.49	**0.18**
SA2499	*gidB*	glucose inhibited division protein B	0.57	**0.36**	0.56
SA2500	*gidA*	glucose inhibited division protein A	**0.55**	**0.34**	**0.51**
Cell wall related genes					
SA0423		hypothetical protein, similar to autolysin	0.58	**0.20**	**0.20**
SA0793	*dltA*	D-alanine-D-alanyl carrier protein ligase	0.63	**0.22**	**0.24**
SA2100		hypothetical protein, similar to autolysin E	**0.46**	**0.32**	**0.30**
SA2354		hypothetical protein, similar to acyltransferase	0.73	**0.37**	**0.47**
Membrane bioenergetics (electron transport chain and ATP synthase)					
SA0910		hypothetical protein, similar to quinol oxidase polypeptide IV QoxD	**0.41**	**0.20**	**0.12**
SA0911	*qoxC*	Quinol oxidase polypeptide III QoxC	**0.34**	**0.19**	**0.14**
SA0912	*qoxB*	Quinol oxidase polypeptide I QoxB	**0.37**	**0.21**	**0.11**
SA0913		hypothetical protein, similar to quinol oxidase polypeptide II QoxA	**0.40**	**0.24**	**0.19**
SA0965	*ctaB*	cytochrome caa3 oxidase (assembly factor) homologue	0.57	**0.40**	**0.35**
SA1909	*atpF*	ATP synthase B chain	0.72	**0.38**	0.49
SA1910	*atpE*	ATP synthase C chain	0.79	0.43	**0.38**
SA1911	*atpB*	ATP synthase A chain	0.66	0.43	**0.39**
Metabolism of amino acids					
SA0180		hypothetical protein, similar to branched-chain amino acid transport system carrier protein	0.54	**0.34**	**0.33**
SA2095		hypothetical protein, similar to D-octopine dehydrogenase	**0.45**	**0.49**	**0.33**
Metabolism of carbohydrates					
SA0562	*adh1*	alcohol dehydrogenase I	2.23	1.59	**0.30**
SA0994	*sdhC*	succinate dehydrogenase cytochrome b-558	**0.58**	0.62	**0.35**
SA0996	*sdhB*	succinate dehydrogenase iron-sulfur protein subunit	0.62	0.58	**0.32**
SA2008	*alsS*	alpha-acetolactate synthase	0.73	**0.39**	**0.29**
SA2102		formate dehydrogenase homologue	0.47	0.86	**0.23**
Metabolism of coenzymes and prosthetic groups					
SA0665		coenzyme PQQ synthesis homologue	0.73	**0.38**	**0.54**
SA0666		hypothetical protein 6-pyruvoyl tetrahydrobipterin synthase homologue	0.61	0.34	**0.38**
SA1494	*hemC*	porphobilinogen deaminase	0.65	**0.33**	**0.42**
SA1495	*hemX*	HemA concentration negative effector HemX	0.68	**0.37**	**0.37**
SA1537		hypothetical protein, similar to thiamine biosynthesis protein ThiI	0.65	**0.35**	**0.43**
SA1538		hypothetical protein, similar to iron-sulfur cofactor synthesis protein NifZ	**0.52**	**0.31**	**0.48**
SA1919		hypothetical protein, similar to protoporphyrinogen oxidase (HemK)	**0.44**	**0.39**	**0.39**
Metabolism of lipids					
SA0820	*glpQ*	glycerophosphoryl diester phosphodiesterase	1.11	**0.47**	**0.29**
Metabolism of nucleotides and nucleic acids					
SA0373	*xprT*	xanthine phosphoribosyltransferase	**0.41**	**0.13**	**0.43**
SA0511		hypothetical protein, similar to UDP-glucose 4-epimerase related protein	0.57	**0.52**	**0.33**
SA0927		conserved hypothetical protein	0.75	0.69	**0.22**
SA1043	*pyrB*	aspartate transcarbamoylase chain A	0.92	2.22	**0.05**
SA1044	*pyrC*	dihydroorotase	0.98	**2.06**	**0.03**
SA1045	*pyrAA*	carbamoyl-phosphate synthase small chain	1.05	1.58	**0.07**
SA1046	*pyrAB*	carbamoyl-phosphate synthase large chain	0.87	1.58	**0.08**
SA1047	*pyrF*	orotidine-5-phosphate decarboxylase	0.81	1.65	**0.05**
SA1048	*pyrE*	orotate phosphoribosyltransferase	0.87	1.43	**0.07**
SA1461	*apt*	adenine phosphoribosyl transferase	0.71	0.62	**0.39**
SA1921	*tdk*	thymidine kinase	0.61	**0.50**	**0.39**

continued on following page

TABLE A2 - *Continued*

N315 ORF	Gene	Gene product	Fold change[2]		
			SA137/93A (0.5xMIC)	SA137/93G (0.5xMIC)	SG511 (1xMIC)
SA2027	*adk*	adenylate kinase	0.53	0.31	**0.33**
Pathogenic factors (toxins and colonization factors)					
SA0270		hypothetical protein, similar to secretory antigen precursor SsaA	0.67	**0.51**	**0.13**
SA0276		conserved hypothetical protein, similar to diarrheal toxin	0.84	**0.34**	0.99
SA0519	*sdrC*	Ser-Asp rich fibrinogen-binding, bone sialoprotein-binding protein	0.53	**0.27**	**0.08**
SA0521	*sdrE*	Ser-Asp rich fibrinogen-binding, bone sialoprotein-binding protein	1.74	1.40	**0.32**
SA0587		lipoprotein, streptococcal adhesin PsaA homologue	0.76	1.21	**0.25**
SA0742	*clfA*	fibrinogen-binding protein A, clumping factor	0.80	1.21	**0.22**
SA0746		staphylococcal nuclease	0.99	**0.26**	0.65
SA1645	*yent1*	enterotoxin Yent1 [Pathogenicity island SaPIn3]	1.28	1.30	**0.39**
SA1647	*sem*	enterotoxin SEM [Pathogenicity island SaPIn3]	1.47	1.31	**0.36**
SA2093	*ssaA*	secretory antigen precursor SsaA homologue	0.57	**0.23**	**0.34**
SA2206	*sbi*	IgG-binding protein SBI	0.87	**0.33**	1.30
SA2290	*fnbB*	fibronectin-binding protein homologue	0.49	**0.27**	1.29
SA2291	*fnb*	fibronectin-binding protein homologue	0.51	**0.32**	1.15
SA2353		hypothetical protein, similar to secretory antigen precursor SsaA	0.69	**0.38**	0.78
SA2356	*isaA*	immunodominant antigen A	0.73	0.51	**0.36**
SA2423	*clfB*	clumping factor B	0.81	0.90	**0.17**
Phage-related functions					
SA1798		hypothetical protein [Bacteriophage phiN315]	0.57	**0.38**	**0.36**
Protein modification, folding and secretion					
SA2028	*secY*	preprotein translocase SecY subunit	0.58	0.32	**0.34**
SA2446		hypothetical protein, similar to preprotein translocase SecY	0.87	**0.39**	1.08
Protein synthesis - initation and termination					
SA1504	*infC*	translation initiation factor IF-3 InfC	0.72	**0.40**	**0.53**
SA1920	*prfA*	peptide chain release factor 1	0.47	**0.37**	**0.41**
Protein synthesis - ribosomal proteins and aminoacyl-tRNA synthases					
SA0354	*rpsR*	30S ribosomal protein S18	0.73	**0.48**	**0.29**
SA0486	*gltX*	glutamyl-tRNA synthetase	0.57	**0.35**	0.53
SA0497	*rplJ*	50S ribosomal protein L10 (BL5)	0.48	0.28	**0.32**
SA0498	*rplL*	50S ribosomal protein L7/L12	0.51	0.37	**0.30**
SA0503	*rpsL*	30S ribosomal protein S12	0.48	**0.28**	**0.40**
SA0504	*rpsG*	30S ribosomal protein S7	**0.42**	**0.28**	**0.44**
SA1081	*rpsP*	30S ribosomal protein S16	0.55	**0.52**	**0.31**
SA1471	*rpmA*	50S ribosomal protein L27	0.46	**0.37**	0.59
SA1922	*rpmE*	50S ribosomal protein L31	0.61	**0.48**	**0.38**
SA2022	*rplQ*	50S ribosomal protein L17	0.45	**0.22**	**0.26**
SA2024	*rpsK*	30S ribosomal protein S11	0.44	0.31	**0.30**
SA2029	*rplO*	50S ribosomal protein L15	0.50	0.29	**0.31**
SA2030	*rpmD*	50S ribosomal protein L30	0.53	0.25	**0.32**
SA2031	*rpsE*	30S ribosomal protein S5	0.66	**0.29**	**0.39**
SA2032	*rplR*	50S ribosomal protein L18	0.49	0.25	**0.29**
SA2033	*rplF*	50S ribosomal protein L6	0.43	0.26	**0.28**
SA2034	*rpsH*	30S ribosomal protein S8	0.50	0.26	**0.35**
SA2035	*rplE*	50S ribosomal protein L5	0.47	0.24	**0.26**
SA2036	*rplX*	50S ribosomal protein L24	0.41	0.25	**0.26**
SA2037	*rplN*	50S ribosomal protein L14	0.43	0.22	**0.28**
SA2038	*rpsQ*	30S ribosomal protein S17	0.42	**0.24**	**0.25**
SA2039	*rpmC*	50S ribosomal protein L29	0.41	**0.22**	**0.25**
SA2040	*rplP*	50S ribosomal protein L16	0.53	0.24	**0.31**
SA2041	*rpsC*	30S ribosomal protein S3	0.43	0.25	**0.24**
SA2042	*rplV*	50S ribosomal protein L22	0.58	0.31	**0.31**
SA2043	*rpsS*	30S ribosomal protein S19	0.54	0.28	**0.29**

continued on following page

TABLE A2 - *Continued*

N315 ORF	Gene	Gene product	Fold change[2]		
			SA137/93A (0.5xMIC)	SA137/93G (0.5xMIC)	SG511 (1xMIC)
SA2045	*rplW*	50S ribosomal protein L23	0.46	0.26	**0.36**
SA2046	*rplD*	50S ribosomal protein L4	0.46	0.27	**0.36**
SA2048	*rpsJ*	30S ribosomal protein S10	0.43	**0.31**	**0.48**
SAS052	*rpsD*	30S ribosomal protein S4	0.71	**0.47**	**0.38**
SAS079	*rpsN*	30S ribosomal protein S14	0.51	0.27	**0.27**
Protein transport and binding					
SA0183	*glcA*	PTS enzyme II, glucose-specific, factor IIA homologue	**0.34**	**0.21**	**0.09**
SA0272		hypothetical protein, similar to transmembrane protein Tmp7	**0.47**	**0.33**	0.97
SA0295		hypothetical protein, similar to outer membrane protein precursor	0.65	**0.31**	**0.26**
SA0374	*pbuX*	xanthine permease	0.52	**0.11**	**0.50**
SA0479	*nupC*	pyrimidine nucleoside transport protein	**0.33**	**0.24**	**0.10**
SA0616	*vraF*	ABC transporter ATP-binding protein	0.64	**0.36**	1.46
SA0617	*vraG*	ABC transporter permease	0.92	**0.38**	1.49
SA0688		hypothetical protein, similar to ferrichrome ABC transporter permease	1.26	0.75	**0.32**
SA0689		hypothetical protein, similar to ferrichrome ABC transporter permease	0.88	**0.69**	**0.26**
SA0690		hypothetical protein, similar to ferrichrome ABC transporter ATP-binding protein	**0.44**	**0.52**	**0.37**
SA0794	*dltB*	DltB membrane protein	0.79	**0.26**	**0.31**
SA0796	*dltD*	poly D-alanine transfer protein	0.75	0.24	**0.30**
SA0928		hypothetical protein, similar to cation ABC transporter	0.90	0.90	**0.23**
SA0950	*potA*	spermidine/putrescine ABC transporter, ATP-binding protein homologue	0.62	**0.31**	**0.30**
SA0951	*potB*		0.58	**0.44**	**0.28**
SA0952	*potC*	spermidine/putrescine ABC transporter homologue	0.51	**0.23**	**0.28**
SA1042	*pyrP*	uracil permease	1.16	**2.02**	**0.07**
SA1224		ABC transporter (ATP-binding protein) homologue	**0.48**	**0.35**	**0.36**
SA1979		hypothetical protein, similar to ferrichrome ABC transporter (binding protein)	0.46	**0.55**	**0.32**
SA2094		hypothetical protein, similar to Na+/H+ antiporter	**0.46**	**0.33**	0.81
SA2117		hypothetical protein, similar to Na+/H+ antiporter	0.82	**0.36**	0.76
SA2132		hypothetical protein, similar to ABC transporter (ATP-binding protein)	**0.34**	**0.22**	**0.21**
SA2135		hypothetical protein, similar to sodium/glutamate symporter	0.71	0.65	**0.23**
SA2142		hypothetical protein, similar to multidrug resistance protein	**0.40**	**0.42**	**0.23**
SA2191		hypothetical protein, similar to NirC	0.54	**0.31**	0.67
SA2300		hypothetical protein, similar to glucarate transporter	0.81	0.83	**0.22**
SA2302		hypothetical protein, similar to ABC transporter	1.12	0.83	**0.27**
SA2303		hypothetical protein, similar to membrane spanning protein	1.15	0.65	**0.18**
SA2326	*ptsG*	PTS system, glucose-specific IIABC component	0.64	**0.42**	**0.32**
RNA synthesis - regulation, elongation and modification					
SA0501	*rpoC*	RNA polymerase beta-prime chain	0.81	**0.39**	0.54
SA1041	*pyrR*	pyrimidine operon repressor chain A	0.72	1.28	**0.13**
SA1082	*rimM*	probable 16S rRNA processing protein	**0.40**	**0.31**	**0.32**
SA1083	*trmD*	tRNA-(guanine-N1)-methyltransferase	**0.44**	**0.32**	**0.38**
SA1583	*rot*	repressor of toxins Rot	0.55	0.64	**0.23**
SA1844	*agrA*	accessory gene regulator A	**0.33**	**0.22**	**0.53**
SA2023	*rpoA*	DNA-directed RNA polymerase alpha chain	0.43	**0.23**	**0.25**
SA2089	*sarR*	staphylococcal accessory regulator A homologue	0.53	**0.36**	**0.31**
Sensors (signal transduction)					
SA1843	*agrC*	accessory gene regulator C	**0.51**	**0.27**	0.70
Hypothetical proteins					
SA0121		hypothetical protein	0.64	X	**0.34**
SA0213		conserved hypothetical protein	**0.33**	**0.39**	**0.37**
SA0231		hypothetical protein, similar to flavohemoprotein	0.90	0.90	**0.36**
SA0262		hypothetical protein	0.51	**0.47**	**0.20**

continued on following page

TABLE A2 - *Continued*

N315 ORF	Gene	Gene product	Fold change[2]		
			SA137/93A (0.5xMIC)	SA137/93G (0.5xMIC)	SG511 (1xMIC)
SA0266		conserved hypothetical protein	1.47	1.03	**0.34**
SA0267		hypothetical protein	1.13	0.78	**0.35**
SA0268		hypothetical protein	1.05	0.90	**0.39**
SA0269		hypothetical protein	1.10	1.18	**0.03**
SA0291		hypothetical protein	**0.31**	**0.36**	**0.11**
SA0292		hypothetical protein	**0.26**	**0.23**	**0.07**
SA0406		hypothetical protein	0.70	0.98	**0.35**
SA0499		conserved hypothetical protein	0.54	**0.28**	**0.53**
SA0555		conserved hypothetical protein	0.74	**0.53**	**0.27**
SA0588		conserved hypothetical protein	0.63	1.26	**0.30**
SA0667		conserved hypothetical protein	0.79	**0.21**	**0.40**
SA0739		conserved hypothetical protein	**0.27**	**0.29**	**0.04**
SA0890		conserved hypothetical protein	0.42	**0.20**	0.58
SA0929		conserved hypothetical protein	0.73	0.94	**0.34**
SA0949		conserved hypothetical protein	0.73	**0.31**	0.52
SA1002		hypothetical protein	1.01	**0.55**	**0.20**
SA1049		hypothetical protein	1.11	1.44	**0.13**
SA1056		hypothetical protein	0.62	**0.53**	**0.35**
SA1265		conserved hypothetical protein	**0.49**	**0.19**	**0.36**
SA1275		conserved hypothetical protein	0.64	**0.57**	**0.39**
SA1536		conserved hypothetical protein	**0.55**	**0.35**	0.53
SA1693		conserved hypothetical protein	0.73	**0.60**	**0.35**
SA1840		conserved hypothetical protein	0.62	**0.39**	0.57
SA1912		hypothetical protein	0.55	0.45	**0.33**
SA1985		hypothetical protein	0.53	1.74	**0.20**
SA1986		hypothetical protein	0.49	1.56	**0.19**
SA2050		conserved hypothetical protein	0.59	**0.29**	**0.44**
SA2133		conserved hypothetical protein	0.51	**0.31**	**0.21**
SA2143		conserved hypothetical protein	**0.46**	**0.53**	**0.30**
SA2328		conserved hypothetical protein	0.90	1.38	**0.37**
SA2329		conserved hypothetical protein	**0.26**	**0.22**	**0.21**
SA2355		conserved hypothetical protein	0.80	**0.39**	0.72
SA2371		conserved hypothetical protein	0.69	0.80	**0.29**
SA2372		hypothetical protein	0.58	0.74	**0.34**
SA2443		hypothetical protein	0.84	**0.37**	1.00

[1] Significant changes of gene expression were determined by implementing SAM (significance analysis of microarrays; http://www-stat.stanford.edu/~tibs/SAM/).
[2] Fold change in transcript level indicated as mean of the "median of ratios" compared to control cells. Fold change in bold = classified as "significantly" regulated in this strain by SAM. "X" indicates the deletion of this ORF in the respective strain according to Reipert et al. (2003).
